# Mapping Research in the Obesity, Adipose Tissue, and MicroRNA Field: A Bibliometric Analysis

**DOI:** 10.3390/cells8121581

**Published:** 2019-12-06

**Authors:** João Manoel Alves, Ramon Handerson Gomes Teles, Camila do Valle Gomes Gatto, Vitor Rosetto Muñoz, Márcia Regina Cominetti, Ana Cláudia Garcia de Oliveira Duarte

**Affiliations:** 1 Department of Physical Education, Federal University of São Carlos (UFSCar), São Carlos 13565-905, SP, Brazil; anaclau@ufscar.br; 2 Department of Gerontology, Federal University of São Carlos (UFSCar), São Carlos 13565-905, SP, Brazil; ramonhanderson@gmail.com (R.H.G.T.); mcominetti@ufscar.br (M.R.C.); 3 Laboratory of Biochemistry and Molecular Biology of Exercise, University of São Paulo (USP), São Paulo 05508-030, SP, Brazil; camilagatto@usp.br; 4 Laboratory of Molecular Biology of Exercise (LaBMEx), School of Applied Sciences, University of Campinas (UNICAMP), Limeira 13484-350, SP, Brazil; vitor.munoz93@gmail.com

**Keywords:** obesity, microRNA, adipose tissue, adipogenesis, bibliometric analysis

## Abstract

Recent studies have investigated the control of adipose tissue expansion and inflammatory process by microRNAs (miRNAs). These two processes are of great interest because both are associated with obesity and metabolic syndrome. However, despite the great relevance of the role of miRNAs in obesity and adipose tissue, no qualitative and quantitative analysis on the subject has been performed. Thus, we aimed to examine global research activity and current trends with respect to the interaction between obesity, adipose tissue and miRNAs through a bibliometric analysis. This research was performed on the Scopus database for publications containing miRNA, obesity, and adipose tissue keyword combinations. In total, 898 articles were analyzed and the most frequently occurring keywords were selected and clustered into three well-defined groups. As a result, first group of keywords pointed to the research area on miRNAs expressed in obesity-associated diseases. The second group demonstrated the regulation of the adipogenesis process by miRNAs, while the third group highlighted brown adipose tissue and thermogenesis as one of the latest global research trends related to the theme. The studies selected in this paper describe the expression and performance of different miRNAs in obesity and comorbidities. Most studies have focused on identifying miRNAs and signaling pathways associated with obesity, type 2 diabetes mellitus, and cardiovascular disease. Thus, the miRNA profile for these diseases may be used as biomarkers and therapeutic targets in the prevention and treatment of obesity-associated diseases.

## 1. Introduction

The prevalence of obesity has increased worldwide and reached pandemic levels in modern society [1,2] A recent global burden estimate suggests that over 1.9 billion people are obese or overweight [3], and about 4 million people die each year from complications related to obesity [1]. Although there have been countless efforts to reduce the progress of obesity, in 2025, the worldwide public health costs related to this condition will reach $1.2 trillion [4]. Notwithstanding, epidemiological studies have illustrated the need to understand and discover new molecular targets and efficient therapeutic approaches to control this pandemic. 

Obesity is characterized by an excessive accumulation of adipose tissue, which is associated with impaired health [3]. Both humans and rodents have three main types of adipose tissue: white adipose tissue (WAT), brown adipose tissue (BAT), and beige adipose tissue [5]. WAT is the main lipid storage site and plays an important role in obesity [6]. In addition to the ability to store energy from the diet [7], WAT-releasing peptide hormones (cytokines) [8], exosomal microRNA (miRNA or miR) [9], and other metabolic agents are involved in the regulation of body energy homeostasis [10]. However, periods of prolonged positive energy balance promote the excessive accumulation of triglycerides and morphological changes that trigger an immune response and pro-inflammatory cytokines secretion, leading to low-grade inflammation [11,12]. These conditions are associated with the development of obesity-related metabolic disorders, such as type 2 diabetes mellitus, cardiovascular disease, and cancer [13,14,15]. Therefore, managing the development of white adipocytes and the mechanisms implicated in their remodeling is one of the main future strategies in the control of obesity and metabolic syndrome.

In contrast to WAT, brown adipose tissue is an organ rich in mitochondria and uncoupling protein 1 (UCP1), which functions through the oxidation of triglycerides and glucose for heat generation (non-shivering thermogenesis), thus preserving body temperature and controlling body fat [16,17,18]. Recently, a structure described as brown-like adipocytes in white adipose tissue, named brite or beige adipocytes, has attracted interest [19]. The transdifferentiating process of white adipocytes in brite adipocytes (browning process) has shown important therapeutic benefits for health, mainly against obesity [20].

miRNAs have been considered a target in obesity control [21,22]. They constitute a class of single-stranded, endogenous, small non-condign RNAs (∼18–22 nucleotides) that regulate 30–80% of the gene expression of the human genome [21,23]. Through trigger-based pairing to complementary sequences within the 3′-untranslated region (3′UTR), miRNAs promote gene silencing by inhibition of translation and/or by affecting mRNA stability and degradation. A single miRNA can be targeted by more than 100 mRNAs subtype, and multiple miRNAs can act in the expression of the same transcript [24].

Several miRNAs are associated with physiological processes, including cell proliferation, apoptosis, neurodevelopment, and tissue differentiation [25]. Furthermore, recent works have analyzed the role of miRNAs in the control of adipocyte differentiation [26,27], metabolism [28], and pathological processes, such as obesity [23,29,30,31] and non-alcoholic fatty liver disease (NAFLD) [32]. In humans, obesity-associated low-grade chronic inflammation is regulated by signal transduction networks and influenced by the expression and secretion of inflammatory cytokines, as well, miRNA-regulation is a significant influence in the whole process [33,34,35,36,37]. Additionally, miRNAs can control the process of adipogenesis during obesity, including transcriptional and post-transcriptional factors such as peroxisome proliferator-activated receptor gamma (PPARγ) and CCAAT/enhancer binding proteins alpha (C/EBPα) [27,38]. For example, the miR-143 was identified as an important adipocyte differentiation-regulator in humans and mice [39,40], while miR-375 promotes differentiation of the 3T3-L1 adipocyte cell line due to the up-regulation of C/EBP and PPARγ2 expression [41]. On the other hand, miR-130a improves insulin sensitivity in obesity [42].

In the obese state, some miRNAs are up-regulated in the WAT of obese individuals compared to lean individuals, such as miR-342-3p, miR-142-3p, miR-142-5p, miR-21, miR-146a, miR-146b, miR-379, while others are down-regulated including miR-122, miR-133b, miR-1, miR-30a, miR-192 and miR-203 [23,29,37]. In addition, miR-17-5p, miR-132, miR-134, miR-181a, miR-27a, miR-30c, miR-140, miR-147, miR-155, miR-197, and miR-210 are potential miRNAs related to adipose tissue dysfunction, obesity and metabolic disorders [43]. Altered expression of a few miRNAs has been experimentally verified in humans [23], evidencing the necessity to conduct more studies aimed at understanding the obesity-specific miRNA regulatory networks and its application as a possible biomarker and therapeutic target against obesity.

Bibliometric analysis is an important tool for monitoring trends in the research domain, determining the impact of research funding, and comparing research progress across different countries, institutions and so forth [44]. In the period between 2005–2019, more than 800 articles related to obesity, adipose tissue, and miRNAs were published. However, the analysis of bibliometric variables regarding these matters remains elusive. Thus, this study aims to provide a literature update related to obesity and miRNAs by employing a bibliometric analysis.

## 2. Materials and Methods

The Scopus database was selected to perform a literature search, once it is considered to be a reliable process for acquiring bibliometric indicators, such as abstracts, citations, and other data mostly used in bibliometric studies in the area of medicine [44,45]. Two investigators (JMA and RHGT) independently conducted a search in the Scopus database using the following keywords and Boolean operators: (microRNA OR miRNA OR miRNAs OR miR-1 OR miR-2 OR miR-3 OR miR-4 OR miR-5 OR miR-6 OR miR-7 OR miR-8 OR miR-9) and (obesity AND “adipose tissue” ORadipogenesis. The papers selected were limited to articles and reviews published between 2005 and 2019, written in English and reported in journals related to the fields of Biochemistry, Genetics and Molecular Biology, Pharmacology and Medicine. All documents were retrieved on 17 June 2019, to avoid bias due to daily updates in the database.

To analyze and discuss the results, data were exported from Scopus in the Bibtex, RIS, and CSV formats. VOSviewer, version 1.6.0 software (Leiden University, Leiden, the Netherlands), was used to analyze the relation between the most cited references and to create maps and clusters. GraphPad Prism version 7 and R Studio version 1.1.463 were used for statistical analysis and to create charts.

## 3. General Information

**Figure 1:**
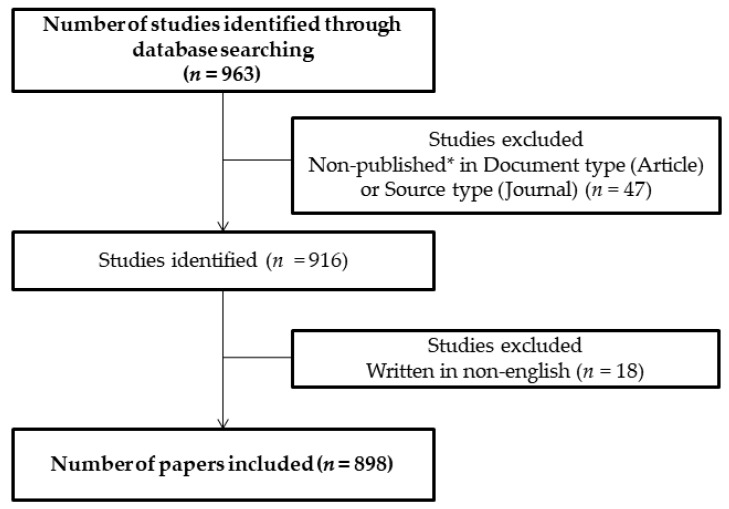
Flowchart of the article selection process used in the study. Notes: * Document and source type include only the articles published in Journals. Conference papers, short surveys, editorials, notes, letters, book chapters, and articles “in press” were excluded.

Initially, 963 studies published in the period from 2005 to 2019 were identified using the keyword combinations. After exclusions, 898 articles were retrieved from the Scopus database, being 670 articles (74.61%) and 228 reviews (25.38%). Together, these articles were cited 25,556 times with an average citation per article of 28.45. The search outcome is shown in the flowchart (Figure 1) followed by the general information concerning it (Table 1). 

**Figure 2:**
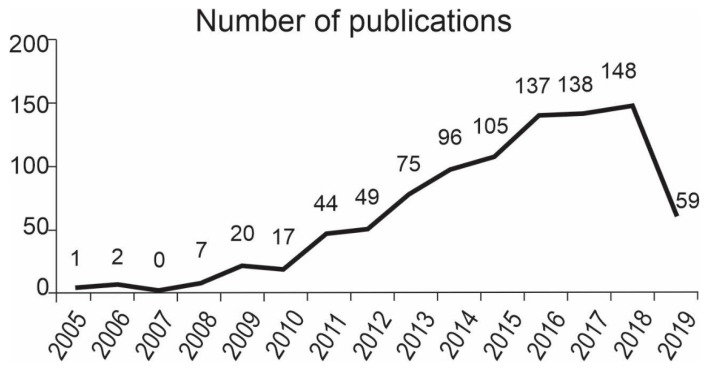
Number of publications on the theme from 2005 to 2019.

After the first study related to obesity, adipose tissue and miRNA was published in 2005 by Hackl and colleagues in the journal *Genome Biology* [46], the number of publications on this theme grew slowly over eight years, and then there was an abrupt increase from 2011. In the period between 2005 and 2019, the number of articles published annually increased from one in 2005 to 148 in 2018, and 59 in the first six months of 2019. The number of documents published per year are shown in Figure 2. Remarkably, 587 articles were published in the last five years, which represent 65.36% of all publications and demonstrate a growing interest in the field in recent years.

## 4. Bibliometric Variables

The bibliometric variables data include information on the number of publication and citations by countries and institutes, the most cited authors, the most cited articles, and sources names (journals). The most productive country was the United States (Supplementary Materials, Figure S1 and Table S1) and the most productive institution was Nanjing Medical University (China) (Supplementary Materials, Table S2), which presented 236 and 26 articles, respectively. The most cited author was “Chenbo Ji”, from the Hospital of Nanjing Medical University, Nanjing Maternity and Child Health Care Institute (China), who produced the majority of the articles, accounting for 14 publications (Supplementary Materials, Table S3). His most cited study is entitled “MiR-148a is Associated with Obesity and Modulates Adipocyte Differentiation of Mesenchymal Stem Cells through Wnt Signaling” [47] with 49 citations.

*PLoS ONE* (IF 2.766, 2018) had the highest number of publications, with 41 articles, and it was the most cited journal with 1,837 citations, followed by the *International Journal of Molecular Sciences* (IF 4,183, 2018) with 33 articles, and *Scientific Reports* (IF 4,011, 2018), with 23 documents (Supplementary Materials, Table S4). The most cited article was “Replicative Senescence of Mesenchymal Stem Cells: A Continuous and Organized Process” [48] published by Wagner, et al. in the *PLo ONE* journal with 630 citations (Supplementary Materials, Table S5). The complete lists with the bibliometric results regarding the theme of obesity, adipose tissue, and miRNA are located in the Supplementary Material.

## 5. Hotspots 

**Figure 3:**
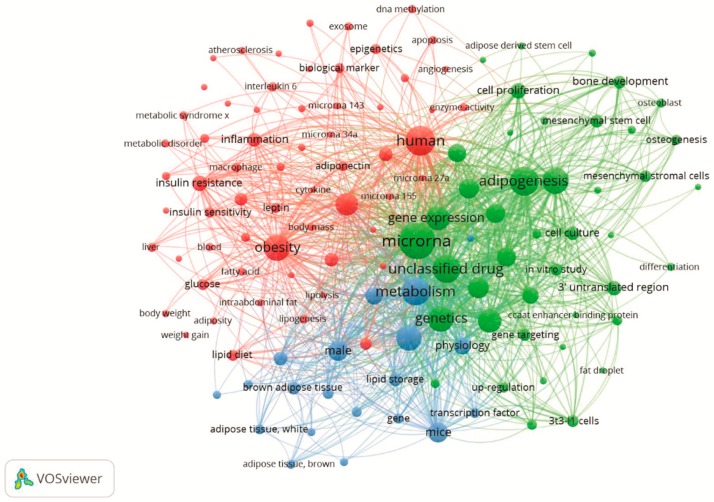
Keywords clusters overview related to obesity and miRNA.

The keywords of the 898 articles were analyzed in the software VOSviewer 1.6.0 software (Leiden University, Leiden, the Netherlands), and a keyword network map was created to establish the trends and current topics within the research area. The keyword network map reflects the research hotspots in the area of obesity, adipose tissue, and miRNA through the weight and strength of the spheres and words [49]. The selection criteria we used was the keywords that appeared more than 10 times in the titles and abstracts, throughout the articles selected. Among the 8,451 keywords, 125 were used and divided into three clusters: red, green, and blue (Figure 3).

The red cluster was titled as “Obesity, inflammation, type 2 diabetes, and cardiovascular diseases: a perspective through miRNAs”. The strongest keywords in this group were human, obesity, and adipose tissue. The green cluster was named “Adipogenic process and miRNAs” and the main keywords were miRNA, adipocyte, and adipogenesis. Finally, in the blue cluster, “MiRNAs in brown adipose tissue control and thermogenesis”, the main keywords were: brown adipose tissue, uncoupling protein 1, and metabolism.

**Figure 4:**
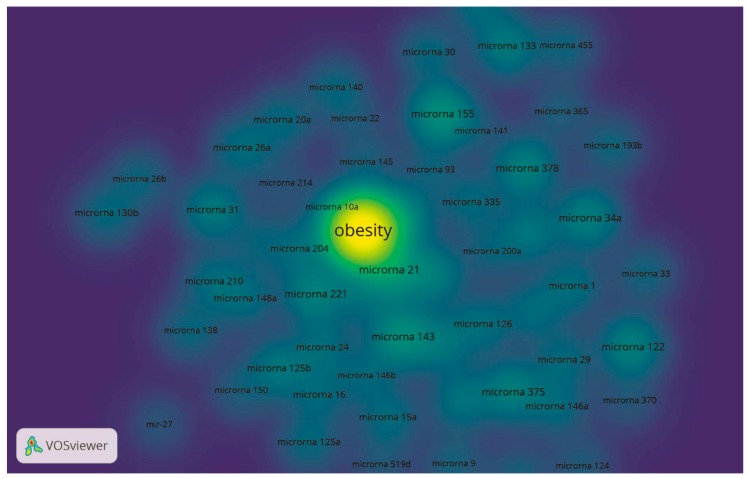
Density map of the most cited miRNAs associated with obesity research.

In addition, the 50 most cited miRNAs were selected based on their occurrence in the articles and are shown in the density map in Figure 4.

### 5.1. Red Cluster: Obesity, Inflammation, Type 2 Diabetes and Cardiovascular Disease: A Perspective through miRNAs

Obesity and metabolic diseases are frequently associated with metabolically dysregulated adipose tissue, as well as liver, skeletal muscle, heart and β-cells alterations. These conditions are supported by studies demonstrating diverse miRNAs being regulated and acting as regulators in the obesity process [28,31]. Here, several obesity-pathological conditions such as atherosclerosis, cardiovascular disease, diabetes mellitus, insulin resistance, and nonalcoholic fatty liver are shown in the red cluster (Figure 2). Among the possible regulatory processes, the presence of keywords such as weight gain, cholesterol, cytokines, fatty acids, glucose, inflammation, macrophages, triglycerides, and tumor necrosis factor-alpha were found in this cluster. 

It is well established that the impairment of adipose tissue function in storage lipid is one of the main causes of metabolic disorders [50]. This process is associated with lipotoxicity, low-grade chronic inflammation, oxidative stress, impaired adipogenesis, and insulin resistance [51,52]. Possibly, the molecules regulating these processes are miRNAs [37,52].

Different miRNAs appearing in the red cluster and Figure 3 are associated with the inflammatory process and cytokine secretion, such as miR-221, miR-378, and miR-155, miR-375, miR-34a, miR-10a, miR-9, miR-146a, miR-146b, miR-125b [53]. On the other hand, miR-146a, an anti-inflammatory miRNA, has also been found (Figure 3) [54]. For example, miR-155 is regulated by several types of immune cells in adipose tissue, such as the toll-like receptor 4 (TLR4), which leads to increased palmitic acid levels that stimulate miR-155 expression [55]. MiR-155 is a potential regulator of the inflammatory process by many pathways, including NF-ΚB and interleukin secretion [56,57]. In obesity, high BMI levels are related to miR-155 high expression in subcutaneous adipose tissue [56], sperm [58], and kidneys [59], suggesting its participation in the inflammatory phenotypes linked with cardiovascular disease, metabolic syndrome, and obesity-related nephropathy.

For example, miR-34a is related to the development of visceral white adipose tissue and inflammation in obese mice [60]. Pan et al. suggest that miR-34a has inflammatory actions and it is a crucial mediator of paracrine actions in adipocyte-resident macrophages. Pan et al. showed that adipocyte-secreted exosomes obtained from miR-34a-*KO* mice were resistant to obesity-induced glucose intolerance, insulin resistance, and systemic inflammation. This was followed by a significant shift in polarization of adipose-resident macrophages from M1 to M2 phenotype by targeting the transcription factor KLF4 in both bone marrow-derived macrophages and adipose depots of obese mice [60]. The same authors showed that miR-34a-overexpression leads to cytokine FGF21 resistance in peripheral tissues, which is associated with the development of insulin resistance and low serum adiponectin levels during obesity [61]. Interestingly, the pathways of KLF4 is a possible link between obesity and bladder cancer. Xiao et al. found that the miR-10 in cells of human bladder cancer promotes cell migration and invasion, suggesting that miR-10b is likely to be a mediator of obesity and bladder cancer, acting as an oncogene in the miR-10b/KLF4/E-cadherin axis and miR-10b/HOXD10/MMP14 axis [62]. For a more detailed discussion about miRNAs involved in the inflammatory process and the oxidative stress occasioned by obesity, see Hulsmans et al. [52].

Unlike inflammation-related miRNAs, such as miR-155, the effects of the absence of miR-146a are well known to promote liver inflammation in miR-146a deficient mice [54]. Interestingly, this miRNA has not been shown to play a role in fat remodeling or weight gain during the development of obesity, but in regulating increased liver inflammation in obese mice [54]. Mechanically, miR-146a regulates the inflammatory network in liver tissue by controlling immune cell infiltration, NF-ΚB activity, and TLR4 signaling, suggesting that its absence leads to an inflammatory response in the liver tissue [63]. miRNAs also have a role in liver disease including NAFLD [32,64]. A study by Estep et al. examining the visceral adipose tissue of NAFLD subjects demonstrated that miRNAs’ expression pattern targets pro-inflammatory adipokines and cytokines such as IL-6, which correlates negatively with expression levels of miR-132, miR-150, miR-433, miR-28-3p, miR-511, miR-517a, and miR-671 [32].

Defects in pancreatic beta cells, and the action of insulin play an essential role in type 2 diabetes, one of the most common metabolic diseases occasioned by obesity [65]. Several miRNAs are involved in the regulation of glycemia and secretion of insulin being up- or down-regulated by obesity, such as miR-30a [66], miR-25 and miR-92a [67], which are involved in transcription, differentiation, and synthesis of insulin. In Figure 3, the occurrence of miRNAs such as miR-150, miR-15a, and miR-103, miR-143, miR-221, miR-26a, miR-17 and miR-125b, miR-126, miR-378, miR-93, miR-26b, miR-27, miR-9, miR-34, miR-375 are related to insulin signaling, beta-cell function and glucose metabolism [68,69]. One of the most studied miRNA, miR-375 is involved in pancreatic B cell regulation, and its response to pancreatic disorders and obesity has been reviewed in previous articles [70]. Early in vitro investigations demonstrated that miR-30a-5p is an essential regulator of beta-cell dysfunction [66]. The increased miR-30a-5p expression is associated with lower glucose tolerance and insulin released by two independent pathways that directly suppress Beta2/NeuroD gene expression [66]. Recently, Jimenez-Lucena et al. demonstrated that circulating levels of miR-30a-5p decrease years before the development of type 2 diabetes [71]. Also, increases in miR-30a-5p expression precede the presence of type 2 diabetes and is higher in individuals with the disease, thus serving as a potential biomarker of the risk of developing type 2 diabetes [71].

Another regulator of type 2 diabetes is miR-143, that acts by phosphorylation of insulin pathways in peripheral tissues. Down-regulated oxysterol-binding-protein-related 8 (ORP8) causes inhibition of insulin-dependent AKT phosphorylation and insulin resistance, a major cause of type 2 diabetes. The general mechanism of miR-143 in insulin regulation is extensively reviewed by Li and Chen [72].

In addition to skeletal muscle, uptake of glucose for the lipogenesis process in adipose tissue occurs in pathways that are insulin-dependent [6]. Previous studies have demonstrated that miR-221 is overexpressed in obese human adipose tissue [73]. Peng et al. demonstrated that miR-221 is up-regulated in white adipose tissue and acts as a pro-inflammatory miRNA involved in the development of type 2 diabetes [74]. The decrease in sirtuin (SIRT) protein expression leads to inflammation and insulin resistance [74]. Interestingly, several miRNAs such as miR-9, miR-181a, and miR-132, are capable of regulating insulin pathways through sirtuin 1 (SIRT1) [75,76], a nicotinamide adenosine dinucleotide (NAD) that regulates energy homeostasis, inflammation, and metabolic disease associated with obesity [77,78]. The miRNAs involved in pancreatic beta cells and diabetes are extensively reviewed in LaPierre and Stoffel [68].

It is noteworthy that cardiovascular disease is one of the main consequences of obesity [79]. In fact, obesity, dyslipidemia, type 2 diabetes and hypertension are important interrelated risk factors that negatively affect cardiovascular health. Considering that cardiovascular diseases remain the predominant cause of morbidity and mortality worldwide [80], it is essential to have a better understanding of the molecular mechanisms that contribute to the pathogenesis of cardiovascular disease and the search for new therapeutic biomarkers for the prevention and treatment of heart disease [80,81]. Thus, miRNAs have attracted increasing attention as shown by the growing number of specific publications related to this theme. mRNAs associated with cardiovascular disease were found in the red cluster, as can be seen in Figure 2 and in Figure 3, including miR-1, miR-34a, miR-122, and miR-126.

The detection of miRNAs and determining their role in WAT-specific depots in obese subjects is essential for the development of new therapeutic targets for this condition. Klöting et al. analyzed 155 miRNAs in obese individuals and showed that 106 had a similar expression in human omental and subcutaneous WAT [43]. However, 16 miRNAs displayed different expression in those that were more common in the omental depot than the subcutaneous depot [43]. Besides, 12 miRNAs were associated with the presence of type 2 diabetes mellitus-related obesity [43]. Thus, obesity promoting changes in miRNAs’ expression pattern in human omental and subcutaneous WAT were shown [43]. In addition, the expression of miR-519d is increased in the subcutaneous fat-depot of obese individuals, which is associated with decreases in target PPARα [82]. Because it is associated with fatty acid oxidation and adipocyte differentiation, PPARα loss may be one of the causes of subcutaneous adipocyte dysregulation and obesity-associated metabolic imbalances [82].

Weight loss is one of the main strategies for the treatment of obesity [83]. Different approaches are utilized for weight loss, such as physical activity, diet, and bariatric surgery [83]. Studies have shown that some miRNAs appear to be regulated when weight loss occurs [84,85]. For example, miR-519d, a human obesity-associated miRNA [82], is decreased in subcutaneous adipose tissue of obese individuals three years after bariatric surgery [84]. In addition, Nardelli et al. demonstrated that after weight loss, miR-519d expression decreased and its target gene (PPARα) increased, suggesting an improvement in the fatty acid metabolism of the subcutaneous fat-depot [84]. Other studies have further investigated the expression profile of miRNAs in weight loss. For example, Ortega et al. demonstrated that surgery-induced weight loss is associated with the marked downregulation of circulating miR-140-5p, miR-122, miR-193a-5p, and miR-16-1 and the upregulation of miR-221 and miR-199a-3p [85]. MiR-122 has been investigated due to its large presence in the liver and involvement in lipid homeostasis [86]. Silencing of miR-122 in mice on a high-fat diet reduces total cholesterol, triglycerides and hepatosteatosis [87], highlighting that miR-122 plays an important role in fatty acid metabolism. Willet et al. showed for the first time, that elevated miR-122 levels in blood circulation are associated with the development of metabolic syndrome and with T2D in humans [86]. Recently, Thompson et al. showed a great increase in miR-122 and miR-199a levels in children with obesity compared to control [88]. Thus, these results suggest that anti-miR-122 therapy may be a promising approach for the treatment of cardiovascular and other metabolic diseases. 

In addition to being a potential biomarker of type 2 diabetes, miR-126 is an independent potential risk factor and diagnostic tool for early prediction of cardiovascular disease [89,90,91]. Interestingly, obesity in sedentary Zucker rats downregulates miR-126 expression and increases its target gene PI3KR2, an indirect negative regulator of VEGF by inhibiting the expression of PI3K [92]. This response induced capillary rarefaction in skeletal muscle, which was prevented by normalization in miR-126 levels and VEGF signaling in muscle in response to 10 weeks of swimming training. Therefore, miRNA-126 should also be considered as an important therapeutic strategy for vascular disorders in the context of obesity [92].

**Figure 5:**
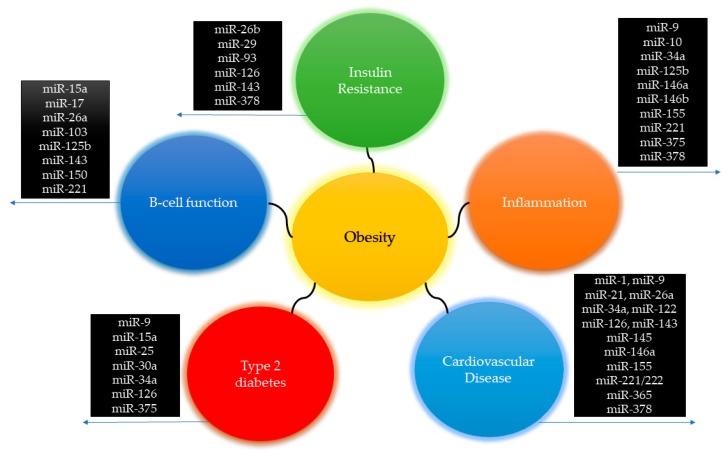
miRNAs associated with obesity-disorders.

Finally, the miR-1 family represents over 40% of all heart-expressed miRNAs, which consist of a subfamily composed of miRNA-1-1, miRNA-1-2, and miRNA-206 [93]. High glucose-induced apoptosis of cardiomyocytes contributes to the development of diabetic complications [94]. In this sense, Shan et al. showed that miR-1 and miR-206 were up-regulated in the myocardium after high glucose, through the MEK1/2 pathway and SRF [94]. The activation of this pathway resulted in suppression of Hsp60 and IGF-1 expression and inhibition of IGF-1/IGF-1R/PI3K/Akt pathway, contributing to glucose-mediated apoptosis in cardiomyocytes [94]. Figure 5 summarizes the obesity-related miRNAs, metabolic disorders, and cardiovascular disease found in this study.

### 5.2. Green Cluster: Adipogenic Process and miRNAs

The differentiation of adipose tissue progenitor cells in new adipocytes is known as adipogenesis [95]. Part of the ability of adipose tissue to expand due to excess energy occurs through the generation of new adipose cells (hyperplasia) [50]. The impairment of this response reduces lipid accumulation in adipocytes, increasing the release of free fatty acids into the circulation and the risk of lipotoxicity in peripheral tissues such as the liver [96]. Recent studies have shown that adipogenesis is a physiological process comprising the potential to be used as a pharmacological target to combat obesity-induced changes [97]. This process is tightly matched by a variety of transcription factors, including PPARγ, the C/EBP family, Sterol 1 regulatory element-binding protein (SREBP1) and mitogen-activated protein kinase 1 (MAPK1) [70,98], as well as by the anti-adipogenic signaling pathways triggered by Wnt, BMPs, transforming growth factor-beta (TGF-β, hedgehog or Rb2/p130 [99,100,101,102]. In addition, miRNAs also regulate the adipogenic process. For example, Xie et al. investigated the pattern of miRNA expression in obesity and the adipogenic process and demonstrated that some miRNAs such as miR-222 and miR-221 are down-regulated, while miR-422b, miR-148a, miR-107, miR-103, miR-30c, miR-30a-5p and miR-143 are upregulated [27]. Several miRNAs that regulate the adipogenic process are present in Figure 3, such as miR-148a, miR-30c, miR-30a-5p, miR-143, miR-221, miR-125b, miR-138, miR-15a, miR-193, miR-21, miR-210, miR-27, miR-29, miR-378. Also, as demonstrated in the green cluster, the adipogenesis process is characterized by the presence of the words: mesenchymal stem cells, cell proliferation, CCAAT enhancer-binding protein, in vitro study and adipogenesis.

MiR-378 plays an important function in the regulation of adipogenesis and oxidative metabolism [98,103]. Huang et al. demonstrated that inhibition of MAPK1 by overexpressing mir-378a-5p in 3T3-L cells promotes adipogenesis and lipid accumulation [98]. Similar to this study, Ishida et al. showed that the expression of miR-378a-5p is inversely correlated with the expression of adiponectin in obese rats, otherwise it positively correlates with the increase of TNF-α [104].

PPARs are a family of nuclear receptors expressed predominantly in adipose tissue [105], which exert effects on energy homeostasis, acting on carbohydrate and lipid metabolism [106], and on the expression of genes involved in the adipogenic process [107,108]. MiR-27a and miR-27b targets mRNA PPARγ, the master regulator of new adipocyte formation [109,110]. Their overexpression inhibits adipocyte differentiation through PPARγ down-regulation [111,112]. Also, a study by Lee et al. showed that miR-130 influences PPARγ expression and reduces the adipogenic process, and lower miR-130 levels are found in obese women [113]. Overall, many miRNAs regulate adipogenesis by different transcription factors and signaling pathways, and there are extensive literature reviews on the subject [30,70,99].

### 5.3. Blue Cluster: MiRNAs in the Control of Brown Adipose Tissue and Thermogenesis

The metabolic importance of adipocytes is mainly described by the classical role of WAT, which stores energy in the form of triglycerides and to secrete adipokines that can control energy expenditure and insulin sensitivity [50]. Conversely, BAT is a key organ in the control of non-shivering thermogenesis in the response to cold and nutritional status. The high amount of mitochondria in BAT can generate heat through UCP1 in the inner mitochondrial membrane by metabolizing glucose and triglycerides [50]. This metabolization is able to clear up to 50% of triglycerides and 75% of glucose from circulation under cold exposure [18,19]. Moreover, under some types of stimulation, brown-like adipocytes are developed within WAT, a process called “browning” or “beiging” of white adipose tissue. These beige cells express UCP1 and show considerable thermogenic capacity [50]. The dynamic control of adipose tissue is not only summarized by its role in the energy control in the adipocytes per se. The homeostatic control of miRNA levels is related to different functions in the differentiation of adipocytes, but the metabolic relevance of miRNAs was showed in adipocyte-specific Dicer knockout mice (ADicerKO) [114,115]. These mice were characterized by lipodystrophy and “whitening” of BAT, dyslipidemia, insulin resistance, and premature aging [114]. In addition, the alterations in their miRNA profile during aging were accompanied by the Dicer levels in white adipose tissue. Interestingly, this miRNA profile in aged mice was rescued by caloric restrictions [116].

These results highlight the importance of miRNAs in the control of fat distribution, adipogenesis, insulin sensitivity, and metabolic control. Thus, using different experimental designs to describe specific miRNAs provides potential approaches to regulating adipose tissue function and treating metabolic disorders. The most cited miRNAs in the studies involved with adipose tissue homeostasis are presented in Figure 3 and will be discussed below.

#### 5.3.1. MiR-193b/miR-365, miR-30, miR-455, miR-378

The miR-193b and miR-365 cluster (miR-193b-365) is highly expressed in BAT and is involved in the adipogenesis of brown adipocytes through the inhibition of runt-related transcription factor 1 translocated to 1 (Runx1t1) [117]. Since the Runx1t1 transcription factor inhibits the adipogenesis of adipocytes, the targeting of miR-193b-365 stimulates the brown adipogenesis in vitro [117]. In addition, the PR domain containing 16 (PRDM16) induces miR-193b-365, facilitating brown adipocyte differentiation [117,118]. However, blocking miR-193b-365 led to inhibition of brown adipocyte differentiation and reduction of adipogenic markers in vitro [117,119]. The in vivo physiological relevance of miR-193b-365 is contradictory to previous in vitro studies, showing that the inhibition of miR-193b-365 in animals does not change the BAT morphology and brown gene markers [120]. 

During adipocyte differentiation, the miR-30 family members are up-regulated, and its inhibition leads to adipogenesis impairment. The miR-30b/c acts through the regulation of the RUNX2 transcription factor and has been described as participating in the regulation of thermogenic genes in vitro [121,122,123]. The miR-30b/c are induced in BAT and subcutaneous WAT under cold exposure, and in response to CL-316,243 (a β3-adrenergic receptor activator) [122]. The overexpression miR-30b/c increases thermogenic gene markers in primary adipocytes through the regulation of receptor-interacting protein 140 (Rip140), a UCP1 silencer [122,124].

MiR-455 is also involved in brown adipocyte differentiation with bone morphogenetic protein 7 (BMP7) as the target [125,126,127]. The miR-455 is a BAT marker in rodents and humans as well as being cold-induced. Mice overexpressing miR-455 in adipose tissue showed enhanced thermogenesis in response to cold and norepinephrine stimulation [125]. The miR-455 targets the adipogenic suppressors RUNX1t1 and Necdin, and consequently activates PPARγ and peroxisome-proliferator-activated receptor γ-coactivator 1α (PGC-1α) [125]. In addition, miR-455 is able to activate AMP-activated protein kinase α 1 (AMPKα1) by inhibiting the hypoxia-inducible factor 1 (HIF1α) [125].

Among the miRNAs involved in adipocyte differentiation, miR-378 shows an opposite effect in brown and beige adipose tissue [128]. Mice overexpressing miR-378 in adipose tissue showed higher brown adipogenesis and BAT mass but reduced inguinal and gonadal WAT mass [128]. Phosphodiesterase Pde1b regulates the cAMP turnover in BAT, but not in WAT, and is shown to be a direct target of miR-378, explaining the possible mechanism by which miR-378 overexpressed mice have impaired beiging of WAT [128,129]. 

#### 5.3.2. MiR-93, miR-155, miR-34a

**Figure 6:**
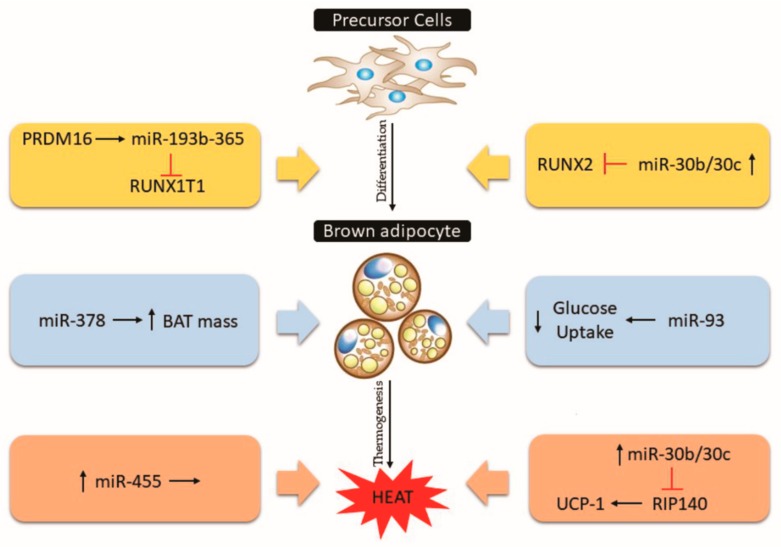
Some miRNAs that control the adipogenic process, glucose uptake, remodeling and thermogenesis in brown adipose tissue (BAT). The action and role of miRNAs are described in the text.

The metabolic capacity of brown adipocytes is related to glucose uptake, lipid turnover, and oxidation [23]. In addition, distinct miRNAs may control the function of these cells. The miR-93 overexpression has been shown to down-regulate the glucose transporter 4 (GLUT4) [130]. In non-obese insulin-resistant individuals, miR-93 is elevated, showing a negative correlation with GLUT4 levels [130]. In addition to its role in the control of adipocyte glucose uptake, miR-93 forms a cluster with miR-106b and regulates the differentiation of mouse embryos [131]. Obese mice showed higher levels of miR-106b and miR-93 in BAT [132]. Figure 6 summarizes the BAT-related miRNAs in the adipogenic process, metabolic responses and thermogenesis.

Nonetheless, the knockdown of miR-106b and miR-93 in brown adipocytes significantly increases the expression of thermogenic genes (*Ucp1, Prdm16, and Cidea*) [132]. Thus, the miR-106b/93 cluster plays a negative role in brown adipocyte homeostasis. Future studies should address the physiological relevance of miR-93 in vivo.

The C/EBPβ protein is an adipogenic transcription factor targeted by miR-155 in vitro [133,134,135]. The interaction between miR-155 and C/EBPβ is suggested as a double-negative feedback loop, where miR-155 inhibits C/EBPβ and premature differentiation; however, C/EBPβ can inhibit miR-155 transcription under pro-adipogenic stimuli [136]. The down-regulation of miR-155 leads to increased browning of the white adipose cell both in vitro and in vivo [136]. In addition, mice lacking miR-155 have shown higher cellular respiration in inguinal WAT under cold exposure [136].

Among the miRNAs with adverse effects on brown adipocytes differentiation, miR-34a suppresses the fibroblast growth factor 21 (FGF21) and SIRT1 regulating the FGF21/SIRT1-dependent deacetylation of PGC-1α [137]. Thus, the inhibition of miR-34a induces the up-regulation of browning genes. The miR-34a was also found up-regulated in the subcutaneous WAT of obese individuals, showing a positive correlation with BMI [137]. In contrast, miR-34a knockout mice have shown increased susceptibility to gain weight under high-fat diet feeding, showing alterations in the macrophage phenotype [138]. More studies are necessary to confirm the contribution of miR-34a in metabolic control in distinct situations of disease. 

Finally, the role of adipose tissue in the control of circulating miRNAs and brown adipose tissue activity and differentiation should be considered as therapeutic targets. Novel studies are needed to complement the data about specific miRNAs targets and their physiological relevance in vivo. In addition, optimizing the cell-specific delivery in different tissues should be addressed by miRNA-based drugs. Future therapeutic miRNA-based strategies capable of increasing adipose tissue energy expenditure may be helpful in the treatment of obesity-associated metabolic disorders. In addition, the endocrine capacity of brown and white adipose tissue has been shown to have physiological importance in the control of metabolic syndrome.

## 6. Strengths and Limitations

Published articles in the area of obesity, adipose tissue, and miRNA were collected in the online Scopus database and were analyzed comprehensively and objectively. However, other databases (Ovid, Pubmed, Web of Science, and Google Scholar) were not analyzed, which could increase the number of papers, authors, institutions, and journals. Furthermore, the low number of publications in languages other than English limits the collection of scientific data from less developed. 

## 7. Conclusions

Here, we have achieved the first bibliometric analysis of the scientific literature related to obesity, adipose tissue and miRNAs. For the period from 2005 to 2019, we quantitatively and qualitatively analyzed global scientific papers on these topics. We systematically summarized the participation of miRNAs in obesity-associated metabolic diseases by clustering the most frequently occurring words into specific themes. In addition, we also reviewed the control of miRNAs in the adipogenesis process in white and brown adipocytes and highlighted those with the most relevance. The results showed that with the global increase in obesity, research on the functions, regulation, and performance of miRNAs in obesity-associated comorbidities has gained prominence. The latest trends point to the presence of exosomal miRNAs secreted by adipose tissue acting on different peripheral tissues, and the participation of miRNAs in the differentiation and activation of BAT. Thus, miRNAs appear as the newest tool to act as biomarkers and pharmacological targets in obesity and metabolic syndromes. Understanding these critical points and how research is being conducted and focused may provide a new approach in the development of new tools to combat obesity in the coming years.

## Figures and Tables

**Table 1:** General information on the papers related to the theme published from 2005 to 2019.

Articles	898
Citations	25,556
Average citations per articles	28.45
Authors	4106
Articles per author	4.57
Coauthor per article	6.98
Collaboration Index	4.66
Sources (journals)	358
Keywords	7261
Collaboration Index	4.66

## Supplementary Materials

The following are available online at https://www.mdpi.com/2073-4409/8/12/1581/s1, Figure S1: Top 10 countries in a number of documents related to obesity, adipose tissue, and miRNA, Table S1: Top 10 countries in the number of citations, documents, average of articles citations, and h-index, Table S2: Main affiliations of authors publishing using the combined keywords obesity, adipose tissue, and miRNA, Table S3: Top 10 authors that most publish on the theme, Table S4: Top 10 journals that most published on the theme, Table S5: Top 10 most cited articles.
