# Supplementary material for: Mapping Research in the Obesity, Adipose Tissue, and MicroRNA Field: A Bibliometric Analysis
Source: Cells. 2019 Dec 6;8(12):1581. doi: 10.3390/cells8121581 (PMC6952878; doi:10.3390/cells8121581)
Supplement: Supplementary file 1 [file cells-08-01581-s001.pdf]

cells

MDPI

Supplementary data

Supplementary Figure S1

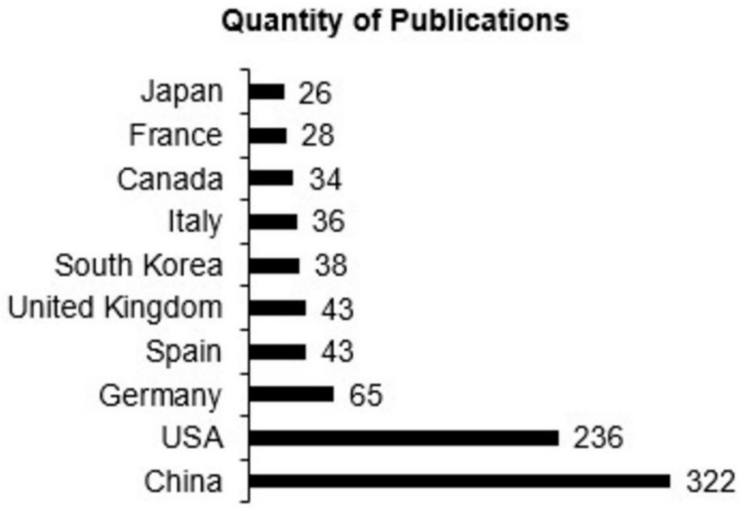

Figure S1: Top 10 countries in a number of documents related to obesity, adipose tissue, and microRNA.

Supplementary Table S1

Table S1. Top 10 countries in the number of citations, documents, average of articles citations, and h-index

Country	Citations	Documents	Average Article Citations	h-index
USA	9,81	236	41.56	52
China	6,631	322	20.59	43
Germany	2,146	65	33.01	20
South Korea	1,481	38	38.97	17
United Kingdom	1,449	43	33.69	21
Spain	1,443	43	33.55	19
Japan	1,407	26	54.11	15
France	1,238	28	44.21	17
Canada	927	34	27.26	18
Italy	720	36	20	12

Supplementary Table S2

Table S2. Main affiliations of authors publishing using the combined keywords obesity, adipose tissue, and microRNA.

Affiliation	Documents	Citations	Average Citations	h-index
Nanjing Medical University (China)	26	616	23.69	14

Ministry of Education China (China)	20	478	23.9	11
Northwest A&F University (China)	20	327	16.35	10
Sun Yat-Sen University (China)	19	393	20.68	9
Inserm (France)	18	534	29.66	12
Harvard Medical School (USA)	18	1,273	70.72	8
Sichuan Agricultural University (China)	17	150	8.82	7
Huazhong Agricultural University (China)	16	344	21.5	10
Instituto de Salud Carlos III (Spain)	15	498	33.2	8
Sichuan University (China)	15	435	29	11

Supplementary Table S3

Table S3: Top 10 authors that most publish on the theme.

Author	Affiliation	Documents	Citations	h-index (2019)
Ji, C	Hospital of Nanjing Medical University, China	14	309	19
Li, X	Sichuan Agricultural University, China	14	144	18
Chen, L	Nanjing Medical University, Nanjing, China	13	328	33
Guo, X	Capital Medical University China, Beijing, China	13	268	40
Scheideler, M	German Center for Diabetes Research (DZD), Oberschleissheim, Germany	12	593	23
Yang, L	Nanjing Medical University, Nanjing, China	11	278	12
Shi, C	Nanjing Medical University, Nanjing, China	10	249	13
Zhu, L	Sichuan Agricultural University, China	10	78	14
Karbiener, M	LKB-Universitãtsklinikum Graz, Graz, Áustria	9	510	13
Li, M	Sichuan Agricultural University, China	9	120	17

Supplementary Table S4

Table S4: Top 10 journals that most published on the theme

ISSN	Journal	h-index	Citations	Documents	CiteScore (2018)	Journal Impact Factor (2018)
**1932-6203**	Plos One	18	1,837	41	3.02	2.766
**1422-0067**	International Journal of Molecular Sciences	9	315	33	4.32	4.183
**2045-2322**	Scientific Reports	12	388	23	4.29	4.011
**1097-4652**	Journal of Cellular Physiology	10	501	22	3.89	4.522
**1879-0038**	Gene	6	388	18	2.60	2.638
**1097-4644**	Journal of Cellular Biochemistry	10	442	18	3.09	3.448
**0006-291X**	Biochemical and Biophysical Research Communications	11	1,087	17	2.69	2.705
**0012-1797**	Diabetes	11	883	14	5.64	7.199
**1071-7323**	Obesity	8	301	13	3.66	3.969
**0307-0565**	International Journal of Obesity	9	388	12	4.82	4.514

Supplementary Table S5

Table S5: Top 10 most cited articles**.**

Author	Document (Source, DOI)	Citations	Document Type	Objective	Main Findings
Wagner, W. et al. [48] (2008)	Replicative senescence of mesenchymal stem cells: A Continuous and organized process. (Plos One, 10.1371/journal.pone.0002213)	630	Article	Analyze morphology, immunophenotype, and differentiation capacity MSC in vitro expansion. To explore mRNA and miRNA expression profiles in MSC.	The replicative senescence of MSC demonstrated functional implications on surfer marker expression and miRNA expression profile. The effect in miRNA profiles can be used in future how therapeutic application senescence of MSC preparations
Rottiers and Nããr. [31] (2012)	MicroRNAs in metabolism and metabolic disorders. (Nature Reviews Molecular Cell Biology, 10.1038/nrm3313)	600	Review	To examine the role microRNAs in metabolic homeostasis, as well as specific microRNAs involved in metabolic disturbers.	miR-122 and miR-33 involved in the maintenance of normal cholesterol and lipid homeostasis. Controlled of insulin signaling and glucose homeostasis by miRNAs. miRNAs associated with cardiometabolic diseases and obesity.
Xie, Lim and Lodish.[27] (2009)	MicroRNAs induced during adipogenesis that accelerate fat cell development are downregulated in obesity. (Diabetes, 10.2337/db08-1299)	367	Article	Investigated the regulation and involvement of microRNAs in fat cell development and obesity.	Presence more 300 mature miRNAs expression during normal fat cell development and adipocytes from obese mice. miR-103 and miR-143 accelerate the rate of fat cell formation. Decreased of patterns expression of miRNAs during adipogenesis in the presence of inflammation.

Huang, Zhao, Xing and Chen. [123] (2010)	MicroRNA-204 regulates Runx2 protein expression and mesenchymal progenitor cell differentiation. (Stem Cells, 10.1002/stem.288)	363	Article	Investigate miRNAs involved in regulation mesenchymal progenitor cells and bone marrow stromal cell (BMSCs) differentiation through modulation of Runx2.	Existence a cross-talk between osteoblast and adipocyte differentiation through miR-204 and miR-211. Expressed both microRNAs during adipogenesis in mesenchymal progenitor cell lines and BMCs. miR-204/211 down-regulated Runx2 suppressing progenitor cells to transform in adipocyte lineage.
Tang and Lane. [95] (2012)	Adipogenesis: From stem cell to adipocyte. (Annual Review of Biochemistry, 10.1146/annurev-biochem-052110-115718)	337	Review	To examine mechanisms involved in the process of adipogenesis.	Overnutrition leads to adipocyte hyperplasia and increased adiposity. Members of family BMP and Wnt were mediators of recruitment of pluripotent mesenchymal stem cells in adipocyte hyperplasia. Transcription factor as CREB, C/EBPα, PPARγ, and SREBP1c coordinately gene expression that produces the adipocyte phenotype.
Lin, Gao, Alarcon, Ye and Yun. [112] (2009)	A role of miR-27 in the regulation of adipogenesis. (FEBS Journal, 10.1111/j.1742-4658.2009.06967.x)	277	Article	Investigated the role of miR-27 in adipogenic differentiation in vitro and genetically obese ob/ob mice.	miR-27a and miR-27b inhibit adipogenesis through downregulation expression of PPARγ and C/EBPα.
Kim, S. Y, et al. [111] (2010)	miR-27a is a negative regulator of adipocyte differentiation via suppressing PPARγ expression. (Biochemical and Biophysical	268	Article	Analyze the inhibitory role of miR-27a in adipogenesis via PPARγ.	Suppressing adipocyte differentiation by miR-27a mediating PPARγ.

	Research Communications. 10.1016/j.bbrc.2010.01.012).				
Karbiener, M, et al. [110] (2009)	microRNA miR-27b impairs human adipocyte differentiation and targets PPARy. (Biochemical and Biophysical Research Communications, 10.1016/j.bbrc.2009.09.098).	265	Article	Investigated of microRNAs expression in human multipotent adipose-derived stem (hMADS) cells.	miR-27 regulated adipogenesis in hMADS. miR-27b was reduced during adipogenesis in human cells. PPARy and C/EBPa are the main target for miR-27b
Kim, Hwang, Bae and Jung. [102] (2009)	MiR-21 regulates adipogenic differentiation through the modulation of TGF-B signaling in mesenchymal stem cells derived from human adipose tissue. (Stem Cells, 10.1002/stem.235)	250	Article	Characterize the role of miR-21 in adipogenic differentiation of human adipose tissue-derived mesenchymal stem cells and signaling pathways involved in this process.	miR-21 is transiently up-regulated during the adipogenic differentiation, mediated through the modulation of TGF-β signaling.
Wang, Q, et al. [101] (2008)	miR-17-92 cluster accelerates adipocyte differentiation by negatively regulating tumor-suppressor Rb2/p130. (Proceedings of the National Academy of Sciences of the United States of America, 10.1073/pnas.0800178105)	248	Article	Analyze functional significance of miRNAs in adipocyte differentiation.	miR-17-92 cluster accelerates adipocyte differentiation. miR-17-92 directly targets and down-regulates the expression of Rb2/p130.
